# Time Matters: The Carbon Footprint of Everyday Activities in Austria

**DOI:** 10.1016/j.ecolecon.2019.106357

**Published:** 2019-10

**Authors:** Barbara Smetschka, Dominik Wiedenhofer, Claudine Egger, Edeltraud Haselsteiner, Daniel Moran, Veronika Gaube

**Affiliations:** a Institute of Social Ecology (SEC), Department of Economics and Social Sciences (WiSo), University of Natural Resources & Life Sciences, Vienna (BOKU), 1070 Vienna, Schottenfeldgasse 29, Austria; b Program for Industrial Ecology, Department of Energy and Process Technology, Norwegian University of Science and Technology, Trondheim 7036, Sam Saelandsvei 7, Norway

**Keywords:** Time use, Sustainable consumption, Carbon footprints, Low carbon activities, Quality of life, Climate change

## Abstract

Mitigating climate change to achieve the goal of staying below 2 °C of warming requires urgent reductions of emissions. Demand-side measures mostly focus on the footprints of consumption. Analysing time use can add to understand the carbon implications of everyday life and the potentials and limitations for decarbonising consumption better.

We investigate the carbon footprints of everyday activities in Austria. We linked data from the Austrian Time-use Survey and the Austrian Household Budget Survey with the Eora-MRIO for 2009–2010 in order to estimate the household carbon footprints of all time-use activities. We introduce a functional time-use perspective differentiating personal, committed, contracted and free time to investigate the average carbon intensity of activities per hour, for an average day and for the average woman and man. We find that personal time is relatively low-carbon, while household as well as leisure activities show large variation in terms of CO2e footprint/h. The traditional gendered division of labour shapes the time-use patterns of women and men, with implications for their carbon footprints.

Further research analysing differences in household size, income, location and availability of infrastructure in their relation to time use is crucial to be able to assess possible pathways towards low carbon everyday life.

## Highlights

• Systems perspective on functional time-use, household consumption and footprints
• Carbon footprint of time use of Austrian households in 2010 estimated
• Average direct and indirect carbon intensity of time use of 1.3 kg CO2e/h
• Gendered division of labour, time- and infrastructure-related constraints important

## 1 Introduction

Meeting the climate mitigation goals agreed upon in Paris 2015 requires substantial and transformative changes to production and consumption in order to achieve absolute and rapid reductions of greenhouse gas emissions globally (Pachauri et al., 2014; Rockström et al., 2017). This aim also necessitates changes in everyday life of people living in industrialized countries and the consumption patterns of households (Akenji et al., 2016; Creutzig et al., 2018; Heinonen et al., 2013a, Heinonen et al., 2013b). Sustainable consumption is a field of research looking at household consumption, its impacts on the environment and the possibilities to influence individual consumption patterns towards more sustainable patterns and levels. The field of sustainable consumption has been criticized as narrowly focused on individual consumer behaviour (Akenji, 2014; Lorek and Spangenberg, 2014; Maniates, 2001), which should be broadened to encompass firstly the relationships between production and consumption, and secondly the relations with human well-being as more appropriate scope than maximizing incomes and consumption (Lamb and Steinberger, 2017; Ropke, 2015).

In the last decade, several multi-regional input-output models (MRIO) have been developed in parallel, which depict the myriad interlinkages in the world-economy and enable attributing resource use and emissions in production along international supply chains to (household) consumption (Malik et al., 2018; Wiedmann and Lenzen, 2018). These models have been applied to a range of issues, for example to household consumption and energy footprints (Jalas and Juntunen, 2015; Min and Rao, 2017; Wiedenhofer et al., 2013), carbon emissions (Fremstad et al., 2018; Gill and Moeller, 2018; Ivanova et al., 2017; Moran et al., 2018; Ottelin et al., 2018; Shigetomi et al., 2014; Wiedenhofer et al., 2017) and various other environmental issues (Kerkhof et al., 2009; López et al., 2017; Shigetomi et al., 2014). In this approach, energy use and emissions along the entire supply chain are usually understood to be ‘indirect’ or ‘upstream’, because they occur during the production process before the household actually consumes the specific goods and services and often account for about half of the total consumption footprint, making their inclusion highly relevant. Households also directly use energy, for example as fuel for cars or for heating. These are termed ‘direct’ emissions. Taken together, direct and indirect emissions account for the entire household footprint of consumption.

MRIO's already allow a very solid systemic analysis of the production consumption linkage. However, it is still a “monetary” perspective on the individual that needs to be extended with other concepts that influence everyday decision-making. Available time - just as available money - governs everyday decision making of individuals concerning living and working arrangements, consumption patterns and means of transportation. Everyday activities need more or less resources and energy. A lack of time can translate into spending more money and more resources, pushing individual and household choices towards less sustainable solutions. Therefore, to understand the carbon implications of everyday life it is necessary to add a time-use perspective.

The functional time-use perspective shows everyday time use as embedded in necessities and constraints due to personal needs, household and family, work and community. This helps to overcome the narrow focus on individual choices and green consumerism, as it emphasizes the relationships between societal arrangements and norms, infrastructures and available services, in shaping and constraining individual activities and related consumption. Focusing on the re/production of household, family, care and community work provides a systemic perspective and allows for a comprehensive analysis (Ringhofer and Fischer-Kowalski, 2016). Time-use analysis enables us to see how the quest to meet all human and societal needs is related to concerns about time squeeze or time prosperity (Southerton and Tomlinson, 2005), which shape the patterns of everyday life (Rinderspacher, 2002; Sullivan and Gershuny, 2018). These relationships have been framed as a vicious cycle of (over)work and consumption, with substantially adverse environmental and social effects (Schor, 2010). Requirements from paid work, available income and communal infrastructure and services further shape individual time-use patterns (Heinonen et al., 2011; Jalas and Juntunen, 2015; Wiedenhofer et al., 2018). Focusing on everyday life patterns can thereby help to develop solutions as well as new perspectives on pathways towards absolute reductions of emissions.

So far, approaches explicitly linking these two research streams are rare. Conceptually, each time-use activity involves the use of goods, services or infrastructure, which means that hours spent on certain activities can be linked to the direct and indirect footprints of consumption involved, either for emissions or the amounts of energy or material involved in performing these activities. Importantly, as is known from footprint studies (see above), large amounts of emissions and resource use occur indirectly along supply chains. Therefore, only focusing on the on-site direct energy/emissions involved in certain activities, severely under-estimates the environmental aspects of time-use activities. A few pioneering studies utilized time-use data and linked it with data on consumption to investigate the footprint of selected activities (see Table 1).

**Table 1:** Overview on research explicitly linking time-use to energy and emissions.

Author	Country	Year of data	Environmental indicator	Time-use covered
Schipper et al. (1989)	USA	1985	Energy	Selected
Jalas, 2005a, Jalas, 2002; Jalas and Juntunen (2015)	Finland	1987, 1998, 2009	Energy footprint	Selected
Brencic and Young (2009)	Canada	2003	Energy	Selected
Druckman et al. (2012)	UK	2005	GHG footprint	All, except ‘volunteering’ and ‘other’
De Lauretis et al. (2017)	France	2010	Energy	Selected
Yu et al. (2019)	China	2008	GHG footprint	All, except “other”

Following the early work by Schipper 1989, these authors (Table 1) investigated the volume and intensity of energy demand or GHG emissions for selected activities. This research already indicates important differences between selected time-use activities. The early work by Mikko Jalas started by linking specific activities (e.g. watching TV, reading, cooking) to their respective energy footprints (Jalas, 2002). He continued investigating their change over the 20 years (1987, 1998, and 2009) with a decomposition analysis, highlighting that beside demographic changes and growing living spaces a higher intensity of consumption, due to “deliberate attempts to save and squeeze time with time saving technologies that range from high speed travel and ready-made frozen food to online ticket services and dating.” (Jalasa and Juntunen, 2015). Angela Druckman expanded on this approach by utilizing a quasi-MRIO model to attribute most emissions footprints to the time-use of the average British person, man and women in the year 2005 (Druckman and Jackson, 2009; Druckman et al., 2012). Most recently, Yu shows the carbon footprint of selected activities for China 2008 (Yu 2019).

In our research, we draw from these pioneers and expand the analysis further towards an analysis of the carbon footprints of the total household consumption in Austria allocated systematically to all functional time-use categories. While the causal relationships between carbon footprints of household consumption, socio-economic conditions and time use are complex, understanding the footprint associated with all daily activities is a first step towards understanding the linkages and feedbacks between time-use patterns and carbon footprints (Wiedenhofer et al., 2018). This is relevant because the constraints and possibilities for change also depend on time, which is limited for everybody and different activities are subject to constraints due to the family, the community and norms about paid work (Ringhofer and Fischer-Kowalski, 2016; Ås, 1978). Herein, we present a comprehensive time-use perspective on the household carbon footprint of consumption for Austrian households in the year 2010. Thereby, we explore how to achieve a full allocation of time use, consumption and footprints and try to answer the following research questions:

• How carbon-footprint intensive are all household activities of everyday life in Austria?
• Are there significant differences between the carbon-intensities of everyday activities and their sum in an average day, taking into account the gendered division of labour between women and men?
• What are the possibilities and limitations for this kind of research for comparative and longitudinal applications, which are necessary to move closer towards the overall research aims sketched out above?

The paper proceeds with an overview of time-use approaches in sustainability research and the operationalization applied herein (Section 2). We then explain the data sources, methods and linking procedures used, covering the Austrian Time-Use Survey 2008/2009 (Statistics Austria, 2011), the Austrian household budget survey 2009/2010 (Statistics Austria, 2013) and the Eora-MRIO (Lenzen et al., 2012, Lenzen et al., 2013) used to estimate the carbon footprints for Austrian households (Section 3). In Section 4, we present the results on carbon footprints per time-use activity and the differences between average women and men in Austria 2010, which are discussed in Section 5 along the research questions. Finally, we draw conclusions on further research needs and for time-use policy.

## 2 Review and operationalization of a time-use perspective on household consumption

### 2.1 The sustainability triangle as a systemic framework

The sustainability triangle symbolizes the interconnectedness of three dimensions of sustainable development, i.e. economic, ecological and social issues (Munasinghe, 1993). This basic systems model shows the interrelations of monetary (economic prosperity), physical (natural resource use) and social (quality of life) dimensions and reveals the unsustainable dynamic of growth inherent in modern society: economic prosperity requires natural resources and results in higher quality of life, which engenders economic prosperity, and so on (Fischer-Kowalski and Haberl, 1998).

Scientific attempts to conceptualize the human impact on the environment link this primarily to human production and consumption. This is important, since the modern industrialized lifestyle and its ever-increasing hunger for resources and energy with its consequences for global environmental change has to be taken very seriously. The ideas on decoupling and downsizing economic activities and societal metabolism (Fischer-Kowalski et al., 2011; Haberl et al., 2017; Swilling et al., 2010) are manifold, intriguing and some of them have already resulted in many activities and effects (Aden, 2016; Bergh, 2017). However, the aspect of quality of life – as the ultimate goal of human activity – is often missing in analysis, as finding appropriate indicators is difficult (Lamb and Steinberger, 2017; Rao and Min, 2018).

We propose that a time-use perspective can provide indicators for quality of life within the sustainability triangle and therefore serve to broaden the analysis of unsustainable patterns of production and consumption (Smetschka et al., 2016; Wiedenhofer et al., 2018). A good quality of life in this time-use perspective includes and emphasizes the aspect of being able to care for all human and societal needs in everyday life and its links to the economic (income) and ecological (carbon footprint) dimensions.

### 2.2 A functional time-use perspective

Functional time-use analysis is a systemic approach that postulates that individuals are part of households and families, the economy and society (Ringhofer and Fischer-Kowalski, 2016). It assumes that individuals “produce and reproduce” these personal, household, economy, and community systems by using their available time, as well as by utilizing and consuming biophysical resources and individual and societal infrastructure. The differing demands and obligations of individuals thereby guide everyday decisions on how to use one's time, how and when livelihoods are earned and how available incomes are spent (Druckman and Jackson, 2009; Jalas, 2005a; Minx and Baiocchi, 2010).

A comprehensive classification of activities and time use needs to address the differences between individual, economic and societal ‘needs’ (Gough, 2017). We can differentiate furthermore, if they necessarily have to be conducted by the individual (e.g. sleep, personal care), or if they can be substituted by someone else's time, for example by consuming services (cleaning, cooking, caring), described as “third person criterion” (Reid, 1934). Finally, most activities rely on a certain biophysical basis, be it infrastructure, housing or goods consumed, which can accelerate, hinder or enhance the individual's time use (e.g. using a car versus walking, reading versus television, availability of appropriate infrastructures, …) (Ås, 1978; Gershuny, 2000; Michelson, 2015; Pentland et al., 1999). For the quantitative analysis conducted herein, we utilize these considerations to define the following functional time-use categories (FTUC) linked to the specific consumption of goods and services (Table 2). Mobility is not a system to be re/produced, but time has to be spent on mobility to enable other activities by allowing people to link spatially distinct activities. It is set apart in the table, as in our research we analysed both, mobility per se and mobility as part of the other time-use categories.

**Table 2:** Functional time use as re/production of systems, time-use categories, encompassing detailed activities and carbon footprints, adapted from (Haselsteiner et al., 2015; Wiedenhofer et al., 2018). For the detailed allocation of all activities and all household consumption footprints, we refer the reader to Supplementary Information 1.

Re/production of system	Functional time-use category	Encompasses activities from time-use survey	And carbon footprints from (examples)
Person	Personal time	Personal care & sleep	Hot water, personal hygiene products, eating, …
Household	Committed time	Household & food preparation; family, care & support	Heating and cooling, cooking, white goods, appliances, furniture, dwelling maintenance, …
Economy	Contracted time	Employment & study	During employment incomes are earned, which enable consumption during all other activities.
Community	Free time	Social activities, politics, culture, leisure	Entertainment activities, sports, socializing, expenditures related to various hobbies, …
Mobility time “enables” other activities by allowing people to link spatially distinct activities		Various forms of travel	Direct emissions from fuels, embodied emissions in transport equipment and infrastructures

The following chapters provide a short description of each of these time-use categories, the main activities encompassed, as well as the major influencing factors on the related carbon intensity of consumption. We outline how the categories are interlinked and affect each other. As time is a limited resource and needs from the different subsystems are conflicting, this interconnectedness can result in time squeeze or time prosperity (Southerton and Tomlinson, 2005). Activities furthermore can vary substantially in how much they rely on consuming goods and services, which socio-technical factors influence these relations and therefore how large the overall carbon footprint of an activity is (see review in Wiedenhofer et al., 2018).

#### 2.2.1 Personal time

The time needed for personal reproduction (sleeping, eating, hygiene, etc.) mostly includes activities, which cannot be delegated to a third party or substituted by services or products and cannot be compressed below a certain point without detrimental effects on the individual. This basic type of time use, therefore, determines the time available for other activities.

The major factors influencing the carbon intensity of the activities in this category are the personal living space per capita, the amount of people sharing a dwelling and the climatic zone (Ala-Mantila et al., 2016; Lenzen et al., 2006; Underwood and Zahran, 2015). Additionally, the degree of parallel consumption, e.g. the use of second homes (Heinonen et al., 2013a) matters. Finally, the heating and cooling technology (means of provision, timing of services, temperature) as well as other material and technological facilities (i.e. changes in washing routines or sophisticated technology in bathrooms, see Shove et al., 2009) available in dwellings make a difference to the footprint related to this functional time-use category.

#### 2.2.2 Committed time

Most people commit a certain fraction of their time to provide for their household and for other persons depending on care, which often are family members. Market-mediated services and products (e.g. preparation of meals, cleaning, care for children, sick and old people) can substitute many of these activities. In spite of technological change and outsourcing, household work is still a relevant time-use activity. The necessity for care work can lead to individual time squeeze, affecting the possibilities for spending time on other activities and consumption decisions (market goods instead of home production, more timesaving electric facilities, convenience food, etc.).

The carbon implications of direct use of energy in kitchen and household activities have changed considerably during the last decades, due to the availability of new technologies (e.g. dishwashers; washing machines; household robots) (Shove, 2003) and in parallel to growing female employment rates in Western industrial countries. Outsourcing can lead to either higher or shared carbon footprints, depending on the availability of public services (for example care and educational facilities), time rebound effects in affluent population groups, lifestyles and parallel consumption (Heinonen et al., 2013b; Ropke, 1999).

#### 2.2.3 Contracted time: employment & study

The main drivers of time-use patterns are the arrangements around employment and education. The amount of working hours and their timing as well as the operating hours of educational institutions and the time required for commuting shape, constrain and enable other time-use activities.

Time squeeze resulting from operating hours, low work-life balances and double burden (mainly of women) can affect the carbon intensity of all other activities through decisions concerning transport means (individual vs. public transport) and consumption patterns (e.g. fast food).

#### 2.2.4 Free time

Time-use studies often focus on free or leisure time, because it is highly variable in its dimension and its consumption intensity and therefore most important to economic and market research.

In addition, it is highly relevant with respect to carbon footprints, since free time can be spent with very high (e.g. weekend city trips by plane) or very low carbon intensity (e.g. socializing with friends). Free time, by its very nature, has an element of free choice. Yet, the carbon intensity of free time can be linked to individual time squeeze and the socio-economic situation. However, public services and communal infrastructure - e.g. green areas, sports and leisure amenities with low costs and carbon emissions and reachable in short time by public transport - shape how we spend free time (Druckman and Jackson, 2009; Jalas and Juntunen, 2015; Rau, 2015). Participative governance structures and active civil society engagement can lead to people spending more time on volunteering and community development, with less carbon footprints and generating useful ideas about sustainable development (Haug, 2008; Schor, 2010).

#### 2.2.5 Mobility time

Many activities described so far require additional time of individuals to move between places where these activities take place. Mobility is typically not a goal per se but is linked to one of the other time-use categories. It has a comparatively small share in the time budget, but a high carbon footprint per hour.

Its environmental implications are closely linked to time squeeze problems as they directly affect modal choice, together with income. Motorized individual transport is the travel activity with the highest carbon intensity. Urban form with differences in density, mixed land use, morphology (polycentric, compact cities) leads to differing carbon footprints (Haselsteiner et al., 2015; Heinonen et al., 2013b; Newman and Kenworthy, 2000; Wiedenhofer et al., 2013). Fast, available and affordable public transport services, true costs of private car use and parking, and short distances between work places, living spaces and community services are key to reductions of energy use and emissions.

## 3 Data and methods

This section describes the time-use survey from which the time-use data were derived and how we assigned consumption expenses and carbon footprint data to time-use activities. We used data from the official Austrian time-use survey and the Austrian household budget survey. We calculated carbon footprints of household consumption, using a consumers-price version of the environmentally extended multi-regional input-output model Eora (Eora-MRIO). We calculated the average carbon footprint of each time-use activity using these three datasets (Table 3).

**Table 3:** Overview of the major data sources used.

	Austrian time-use survey	Austrian household budget survey	Multi-regional input-output model (MRIO), Eora
Temporal coverage	03/2008–03/2009	04/2009–05/2010	2009 and 2010
Population covered	All persons above 10 years	All households	192 countries/economies
Sample size	8234 individuals in 4757 households, sample weighting provided to achieve representativeness	6534 households, sample weighting provided to achieve representativeness	Complete representation of the world-economy in 25–340 sectors, depending on country-level information
Response rate	38%	38%	*Not applicable*
Number of categories	83 categories describing all activities	Study-specific classification of expenditures into 53 categories, derived from detailed COICOP data structure	In the Eora-MRIO, the Austrian economy is represented along 59 consumption categories, additionally 4 categories of direct energy use were estimated.
Source	Statistics Austria (2011)	Statistics Austria (2013)	Lenzen et al., 2012, Lenzen et al., 2013

### 3.1 Austrian time-use survey 2008/2009

The most recent time-use survey in Austria covers the period from March 2008 to March 2009 (Statistics Austria, 2011). The number of respondents was *n* = 8234 persons aged 10 and above living in 4757 private households. Activities were recorded in a time diary for one full day in respondents' own words in 84 time slots ranging over 15 min between 5 a.m. and 11 p.m. and over 30 min for the remaining time. The time diaries were coded into 427 activities. The time survey focuses on time use per “normal” day individual. The time-use data for holidaymaking are not consistently available. In order to link these data to consumption data, the time-use data was extrapolated to an annual, national level. As a first step, the 427 time-use activities were aggregated to 83 time-use activities (20 activities for the 4 functional time-use categories plus 63 different mobility activities as available from the survey).

The daily time spent was summarized over activities per individual and day *a*_*n*_ to calculate time use for an average day, differentiating between weekdays *j* and weekend days l. With the person weight *pw*_*i*_, corresponding to the number of individuals from the total population represented by the particular respondent *resp* and the yearly occurrence of the two ‘types’ of days *yfac*_*j*_ and *yfac*_*l*_, the annual time use was then summarized to the national level, where *T* = 65 billion h spent per year on 83 time-use activities (Eq. (1)).

(1) $$T=\sum_{i=1}^{8234}\mathrm{res}*{\mathrm{pw}}_{i}*\left(\frac{{\mathrm{resp}}_{j}*\sum_{n=1}^{N}a_{n}*{\mathrm{yfac}}_{j}}{\sum_{j=1}^{J}\mathrm{resp}}+\frac{{\mathrm{resp}}_{l}*\sum_{n=1}^{N}a_{n}*{\mathrm{yfac}}_{l}}{\sum_{l=1}^{L}\mathrm{resp}}\;\right)$$

### 3.2 Austrian household budget survey for 2010

The household budget survey contains data on monetary consumption patterns *y*_*i*_ of 6534 Austrian (1246 Viennese) households randomly chosen in 2009 and 2010 (Statistics Austria, 2013). Statistics Austria provides a weighting procedure for the data to correct for sampling design and non-responses and to provide calibration of the sample against the distributions found in the national micro-census to ensure the survey sample was representative. Participation in the survey was voluntary with a response rate of 38%. The survey data is classified along the standardized UN Classification of Individual Consumption by Purpose (COICOP) and in total represents the total monetary final demand by all Austrian households. This total household final demand is also reported in the national input-output tables and therefore in the Eora-MRIO, which allows us to combine these two data sources. For this study, not only COICOP level carbon footprints were estimated, but a specific grouping of expenditure and footprints along 53 consumption categories directly attributable to the time-use categories had to be assembled from the detailed data.

### 3.3 Estimating direct and indirect carbon footprints of Austrian household consumption

To estimate footprints of household consumption, we utilize a consumers-price version of the multi-regional input-output model Eora (Eora-MRIO). The MRIO covers 192 countries of the world and uses EDGAR data on carbon emissions and IEA data for energy use (Lenzen et al., 2013, 2012). MRIOs are usually constructed using so-called basic or producers' prices. This is achieved by removing the valuation layers (taxes, subsidies, margins and transport costs) from the national input-output tables. The MRIO also contains a vector of household final demand for Austria (and for 191 other countries), in basic prices. At the same time, the household budget survey is composed using purchasers' prices, which include the above valuation layers. To link both data sources, different approaches are used in the literature.

Herein, a consumers-price version of the Eora-MRIO was used, which included all valuation layers. We first calculated the indirect Austrian household footprint using the Eora-MRIO, which contains the Austrian economy and therefore household consumption along the CPA2002 classification. However, the household budget survey is classified in the COICOP nomenclature. Statistics Austria provided a concordance matrix between COICOP and CPA2002. This allowed us to re-allocate the total household footprint calculated in the Eora-MRIO to the COICOP classification. We then used the detailed budget survey to calculate the monetary share of each household's consumption in total final demand, to allocate the national total to each household. Thereby we can ensure that the entire household footprint calculated in the MRIO is allocated among the Austrian households from the budget survey. For this purpose, we apply the standard input-output identity of the Leontief inverse (**I – A**)^−1^, which allows us to estimate the CO2e emissions **q**, which were emitted during the production of the goods and services **F** required to satisfy a specific level of final demand **y**, for each sector*I*(Eq. (2)):

(2) $$q=F*{\left(I-A\right)}^{-1}*y_{i}$$

To account for the direct energy use and emissions from household activities, the household budget survey provided the starting point, because it contains data on the annual expenditure on gasoline, diesel, heating oil, natural gas and electricity per household. Calculating the physical quantities of direct energy use based on expenditure data was done via energy prices for private consumers by each energy carrier for the years 2009/2010 (Statistics Austria, 2012). No regionalized price data is available and national values had to be used. The fuel price dataset contains a distinction between regular gasoline and premium gasoline (octane ratings of 95 and 98) that is not present in the budget survey, which only contains consumption of gasoline in general. Therefore, data on annual total sale of each fuel was used to arrive at an average Austrian gasoline mix and therefore average price of gasoline over these three different types, which was then used to calculate physical household gasoline consumption. To arrive at the energy content of total gasoline use the same data that Statistics Austria uses for their energy accounts (OECD/IEA, 2005) has been applied.

Austria specific carbon emission factors per fuel were sourced from the IPCC emissions database (IPCC-EFDB, 2007). While conceptually energy and emissions from electricity are often seen as part of direct energy use, these are actually part of the MRIO, where energy use, transformation losses and emissions are allocated to the electricity supply sector and therefore do not require additional calculation steps because they are covered in the MRIO multipliers described above.

Overall, we find good agreement between the total carbon footprint of Austrian households as presented in Steininger et al., 2018, who estimate a total footprint of 83 million t of CO2e for the year 2011; while herein we find 87 million t of CO2e for 2009/10.

### 3.4 Estimating carbon footprints of time-use activities: linking time, household consumption and carbon footprints

Both time-use and budget surveys are not unambiguously compatible and no official concordance between both is available. Therefore, building on recent work by other researchers, we had to allocate household expenditure to specific time-use activities (Druckman et al., 2012; Jalas, 2002; Jalas and Juntunen, 2015). Difficulties arise from the effort to combine three data sets differing in sample (age limits, range), units (persons, households, nations) and goals (everyday living, monetary expenditure, environmental pressures). We met these challenges as follows: From the time-use survey data, a representative sample of the time use of 8234 persons in Austria is available. Based on reported survey weights, we calculated the total hours spent by all people in Austria, by days and by gender. From the household budget surveys, data is available on the total household consumption in Austria and using the Eora-MRIO we can estimate the direct and indirect carbon footprint associated with all household expenditures. The results show the volumes of total hours per activities and total CO2e for all COICOP categories. As a simple validation, one can divide the total household footprint of 87 million t CO2e/y by the total time spent, which is 65 billion h/y, resulting in an average carbon intensity of 1.3 kg CO2e/h. For the more detailed assessment, we allocated each of the 53 expenditure/consumption categories to one or more time-use activities, resulting in an 83 × 53 concordance matrix of the time-use categories for activities and the consumption categories from the household budget survey (see Time-Consumption Matrix in the supplementary information 1). Using this procedure, we arrive at the same average carbon intensity of 1.3 kg CO2e/h.

As in the two comparable studies (Druckman et al., 2012; Jalas, 2002) this assignment is to some degree arbitrary and was based on literature research and group discussions within the project team. We used Druckman et al. as a starting point for the Time-Consumption Matrix but agreed on a few deviations from their allocation table, mainly due to differing data classifications and availability. While Jalas focused on specific activities, Druckman et al. allocated most of the household footprint to activities. Building on this, we also assigned all expenses and thus 100% of the household carbon footprint to activities.

The following assumptions were also applied: we allocated some of the basic living costs to an array of activities comprising this consumption, i.e. time-wise non-specific living costs, such as costs for electricity, water, etc., were allocated to all activities inside one's home including Sleep. We allocated tobacco consumption evenly across all activities, assuming that smoking happens throughout the day. We allocated alcoholic beverages to eating and drinking. Because employment is the very activity, which enables the production of goods and services, and where household incomes are earned, no household expenditures and carbon footprints can be directly and clearly allocated to the time spent in paid work, because all work-place emissions are part of the supply chains of all goods and services consumed during all other activities. Although time-use data only represent mostly “normal” days, not holidays, we did not want to exclude catering and accommodation services as they represent a significant amount of expenditure and carbon footprint. Here we opted to allocate the carbon emissions to free time activities, which can be spent outside the home. Each assigned consumption category adds up to the total consumption expenses per activity category. If a consumption category is assigned to more than one activity, the consumption expenses are distributed between all assigned activities based on their relative time use.

### 3.5 Limitations, uncertainties and assumptions

#### 3.5.1 Time-use survey

The method of time diaries and the time-use survey in particular have several shortcomings that can affect our results. Importantly, the survey data lacks information on holiday making and specific long-range (i.e. air) travel and does not provide consistent long-term observations on time use of the respondents. Additionally, a low spatial resolution hinders potential additional analysis, for example of the location of households. Unfortunately, the survey lacks information on the income levels of respondent and/or households, hindering explicit analysis of the dependency of time-use patterns and therefore consumption footprints on available money. As a strong point for our purposes we see the data on travel and the high n (sample is 0.1% of total pop.). Time diaries are the most widely available and agreed-upon type of data for analysis of time use (Eurostat, 2009; Gershuny, 2000).

#### 3.5.2 Multi-regional input-output approach

The input-output methodology relies on three main assumptions: firstly, constant prices across industries and consumers, secondly the proportionality of demand and production (the Leontief production function), and thirdly, homogenous sector/product mixes. The constancy of prices and homogenous product mixes are a generally applied and accepted part of the methodology for studies on environmental issues (Lenzen, 2001). Problems could arise if fuel and electricity prices have substantial regional variations. However, as Austria is a relatively small country, such variations are deemed minimal. The proportionality assumption can furthermore affect consumption-based estimates of the scaling between footprints and overall expenditure/income levels, however this is an unresolved issue for the entire field (Girod and de Haan, 2010; Heinonen et al., 2011; Kok et al., 2006; Min and Rao, 2017).

Building a multi-regional input-output model requires major data and time efforts and is often constrained by the strongly differing number of sectors in each country's input-output table and by the lack of standardized datasets on energy use and carbon emissions per sector (Lenzen et al. 2012, 2013; Malik et al., 2018; Moran and Wood, 2014; Owen et al., 2014). Some deviations from national level data sources for energy and emissions do occur in this study, where for example Statistics Austria reports 5–8% larger energy use and emissions than the Eora-MRIO includes for Austria. This is partly due to the fact that Eora uses IEA energy data and Edgar emissions data and as Moran and Wood (2014) have pointed out, carbon accounting lacks standardisation in important areas. While many developed countries report GHG emissions inventories, which are detailed by emission source, they do not report the responsible sector. Therefore, when MRIO databases integrate these national emissions inventories, assumptions must be made when attributing total territorial emissions among sectors. How this sector-wise allocation is done differs between MRIO databases (Moran and Wood, 2014; Owen et al., 2014). While the findings of Moran and Wood (2014) show that national total footprints are relatively robust against sector-wise allocation of territorial emissions across all major MRIOs, for more detailed studies such as ours, sector-wise allocations become more important. Improving accounting conventions like this is an active topic among MRIO creators. As questioning the methods used for this allocation along sectors is beyond the scope of this study, we simply note this issue and assume that accounting conventions will become more robust in future years.

#### 3.5.3 Combining time-use survey data with household budget survey data

Due to data restrictions mentioned above, time-use and household budget data could only be combined for the average Austrian individual and household, mainly because of the lack of information on income levels for the time-use survey. Time-use data include all persons aged above 10 years. The Household Budget Survey however includes all household expenditures, no matter if they are spent on children <10 years or not. While technically it would be more correct to exclude the footprints of children <10 years, the data does not allow for such a differentiation of expenditure/consumption. We divided the total hours spent in Austria by persons older than 10 years to obtain the time-use profile of an average person. We calculated carbon footprints (CO2e) for the total expenditure of all Austrian households. We then divided these carbon footprints per budget category through the total hours of activities attributed to these budgets, resulting in an average CO2e intensity per hour for each activity.

Differences in carbon footprints were calculated along different time-use patterns, for example for average women or men. The important variations in consumption, which result from household income, cannot be analysed here, as time-use data did not refer to income data. Being aware of the pitfalls of ecological fallacy we summarize the aim of this study: Combining time use of an average person with the average carbon footprint of an activity can only be a means to get better knowledge on constraints and possibilities of change in everyday life as a whole, not a tool to analyse causal links on isolated items.

## 4 Results

### 4.1 Carbon footprints of Austrian households: total, per functional time-use category and per activity

**Fig. 1:**
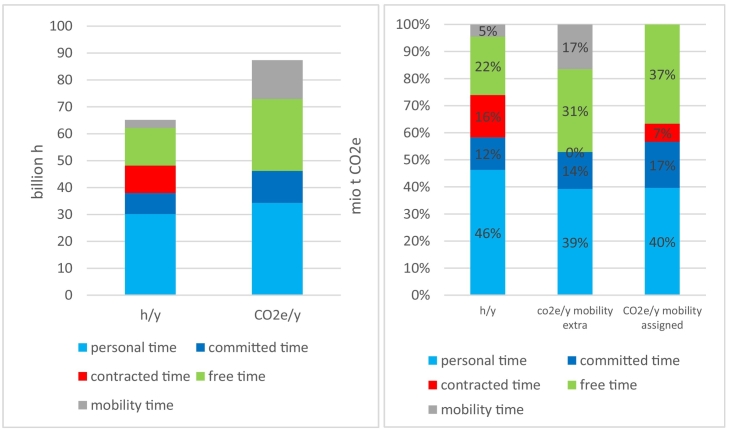
a + b: Total time use by activity in billion h/y and total carbon footprint of households by activity in million t CO2e/y in absolute values (1a) and as shares of the total (1b) for Austria in 2010.

Fig. 1a shows that the total calculated hours of Austria's population in 2010 amount to 65 billion h. This time was spent for personal, committed, contracted or free time purposes, i.e. the four functional time-use categories introduced in Section 2.1. The total Austrian household footprint estimated in this study is 87 million t CO2e, which is approximately half of the national total footprint, the remainder being investments, exports and government consumption (Steininger et al., 2018). Only the total carbon footprint of household consumption was allocated to time-use activities.

When we look at the composition of time use and carbon footprints (Fig. 1b), we find that for the average Austrian, personal time accounts for 46% of time use but only 40% of the carbon footprint. Free time, in contrast has a share of 22% of time use while it amounts to 37% of the carbon footprint. Not surprisingly, we find that mobility is a crucial factor. It consumes 5% of total time use in Austria and is responsible for 17% of the carbon footprint; hence, the time used for mobility has a particularly high carbon intensity. Since mobility is associated with other activities, it can be directly assigned to these activities (right column in Fig. 1b), thereby increasing the carbon intensity of the different activities. Assigning all work-related commuting to contracted time amounts to 7% of the total household carbon footprint. Mobility and free time together add up to nearly half of the carbon footprint of households.

**Fig. 2:**
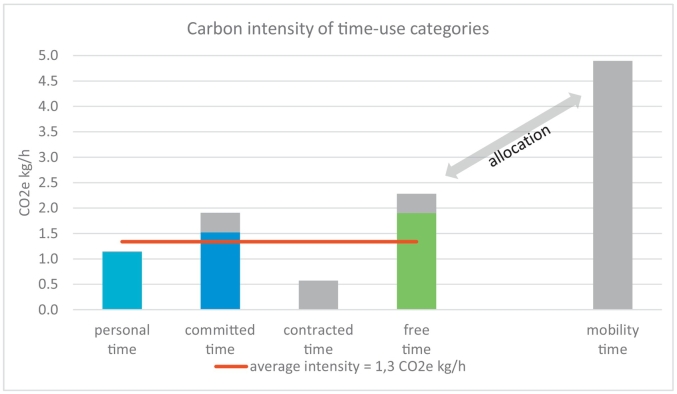
Average carbon footprint intensity of functional time-use category, with mobility allocated (bars on the left, incl. grey component) versus all mobility time and footprint extra (right hand side, grey bar). Note that contracted time does not have a carbon intensity, as all emissions at the work place are allocated to the carbon footprint of goods and services consumed during other activities.

Dividing total CO2e by total hours results in an average carbon intensity of 1.3 kg CO2 equivalents per hour and person in Austria (Fig. 2). Mobility time with 4.9 kg CO2e/h has the highest carbon intensity. Free time has the second highest hourly carbon footprint, with 1.9 kg CO2e/h, while personal time has the lowest footprint per hour, with 1.1 kg CO2e/h. Although all emissions related to housing (heating, cooling, electricity use) and goods consumed in households have been allocated to this activity, the carbon footprint per hour is still relatively low, simply because this is also the most time-consuming category, where 79% of it amounts to sleeping time. When allocating all mobility time and the respective footprint to the time-use categories ‘enabled’ by individuals being mobile, the results change slightly: the carbon footprint per hour of free time increases by 17%, while the footprint of committed time increases by 20%. Personal time however is unaffected, because these activities happen within the home. Allocating all work-related commuting to the time spent in paid employment results in 0.6 kg CO2e/h.

One important result of looking through the time-use lens is this: activities, which take many hours and do not rely on energy and emissions intensive supply chains, have less carbon footprint than others do. Mobility time has by far the highest carbon intensity, because high amounts of energy are used in a short time.

**Fig. 3:**
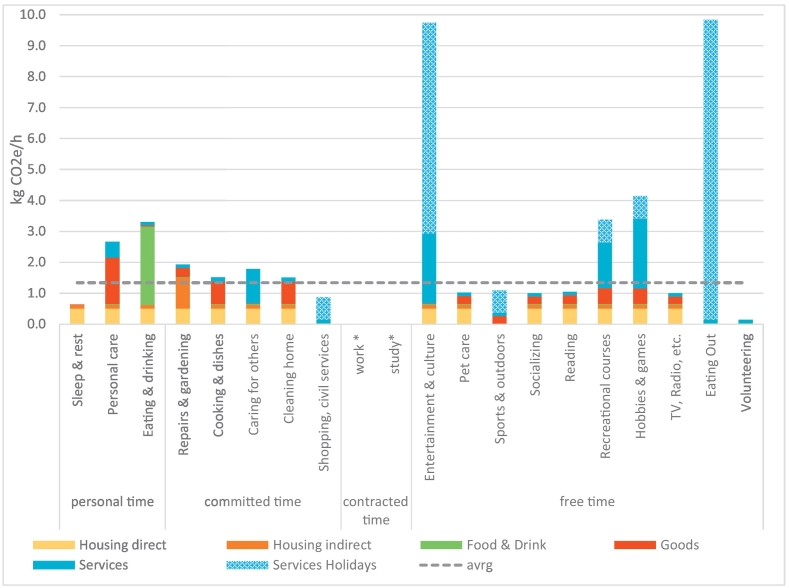
Carbon intensity of time-use activities in detail. Note that this figure excludes mobility time and footprints. The time-use categories work* and study* have no carbon footprints because this is the time when incomes are earned, which are spent during other activities. Expenditures on holidays, catering and accommodation services are assigned as “Services Holidays” to Shopping, Entertainment, Sports, Recreational courses, Hobbies and Eating Out.

The carbon intensity of time-use activities differs within the functional time-use categories. Fig. 3 shows a further disaggregation of activities (excluding time spent and emissions for mobility). All activities inside one's home show the basic living footprint at the bottom (housing direct and indirect). Eating and drinking include high carbon emissions resulting in a high carbon intensity 3.3 kg CO2e/h. The biggest differences stem from the amount of goods and services consumed for different activities. Personal time has an average high intensity due to eating and all goods needed for daily living. Activities associated with family and household do not vary much from the average intensity of 1.3 kg CO2e/h, while free time has a high level of variations between the specific activities. Activities at home using little resources (e.g. socializing) or long-living goods (e.g. TVs) are below the average intensity around 1 kg CO2e/h.

Activities involving a high amount of resources are linked to expenses for Entertainment, Recreational courses and Hobbies. We assigned the expenditures on holidays, catering and accommodation services as extra “Services Holidays” to activities spent outside the home: Shopping, Entertainment, Sports, Recreational courses, Hobbies and Eating Out, which can lead to carbon intensities over 9 kg CO2e/h. It is difficult to assign the carbon emissions stemming from the high amount of money we spend on the catering and accommodation services and holidays to time-use activities. However, the data show that excluding it from everyday life would imply losing sight of an important amount of carbon emissions. Reducing the expenses spent on holidays and in gastronomy to zero leads to an average carbon intensity of 1.17 kg CO2e/h. Thus, it appears that these expenses amount to 16% of carbon emissions.

### 4.2 Carbon footprint of time use per day and by gender

**Fig. 4:**
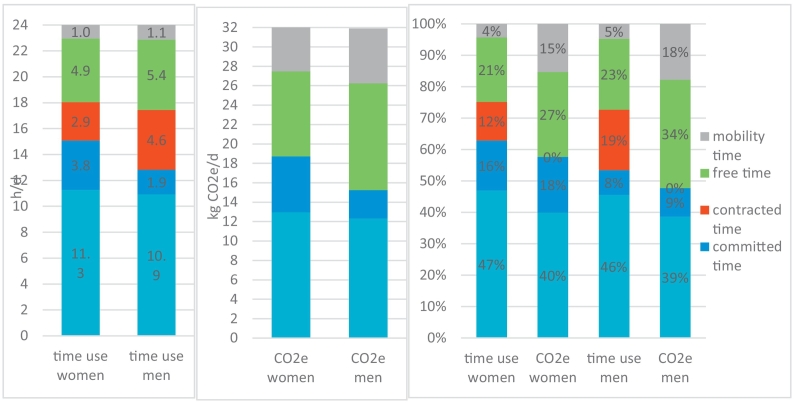
The carbon implications of the average day of women and men in Austria, in hours per day (a), the respective average carbon footprint in CO2e per day (b) their compositions (c).

As time is a matter of concern to everybody in daily life, which is limited and equally divided for all humans, we also analyse an average day. In Fig. 4, we show how many daily hours an average woman or man in Austria spends within each category. A traditional gendered division of labour is clearly visible, as women spend double the time with committed activities than men. Men spend more time in employed work than women, who largely work part time in Austria. Calculated across all differences in age, workload and commitment, men have 30 min more free time per average day than women do.

Calculating the average daily carbon footprints of Austrian women and men shows the impact of the different carbon intensities of each time-use category. Women spend more time with personal and caring time, the latter having a comparatively high carbon intensity, while men spend more time with free time activities and mobility. Again, the results reflect the traditional division of labour here, as women spend more time with caring activities and men with employed work and with sports, hobbies and watching TV. The carbon footprints of free and mobility time amount to 42% (women) or 52% (men) of the average daily footprint.

## 5 Discussion of results

### 5.1 Comparison with prior research

So far, little research has been linking total carbon footprints of households to all time-use activities. The noteworthy exceptions are the study of Druckman et al. (2012), which showed the carbon footprints of time-use activities of average British women and men and the study of Yu et al. (2019) calculating carbon footprints of time use in China. Interestingly, our results for Austria for the year 2010 corroborate previous work on the UK for the year 2005 (Druckman et al., 2012), despite differences in years as well as data and model used. While Druckman et al. reported the average carbon intensity of time use in the UK to be 1.2 kg CO2e/h in 2005; we find 1.3 kg CO2e/h for Austria in 2010. We also re-analysed Druckman's results along our functional time-use categories, where we find similar carbon intensities for the functional time-use categories, insofar as eating and committed time have a higher intensity than most free time activities excluding holidays (see Supplementary Information 2).

However, when disaggregating the results and comparing specific activities, we find a number of differences. This is due to the fact that we included all activities of an average person - systematically grouped into four functional time-use categories - and fully allocated all household expenditures to these activities. As we did not exclude any consumption items, we had to allocate them to time-use activities. Basic living costs calculated as carbon emissions stemming from living in large, well-furnished, heated/cooled and lightened flats therefore raise the carbon footprint of all activities at home and are allocated to Sleep as well. The emissions per hour sleep are high because we divide the emissions of basic living within comfortable flats equally among all hours spent there, notwithstanding the fact that heating maybe lowered at night and lights will be switched off. Reduced or increased sleeping hours will not change the carbon footprint of an average basic living standards. More importantly, it is changes in heating/cooling or lighting practices which make a difference. Druckman found a higher intensity for Personal care, probably allocating more of the expenses to Personal care than to Sleep. We allocated expenditure for alcoholic drinks to Eating and Drinking, and tobacco equally to every person's daily activities. Druckman still shows a higher footprint for Eating and Drinking than we estimate in Austria. We did not follow Druckman in excluding Volunteering, but regard it as a free time activity with very low carbon footprints.

In our work, we further decided to include the expenditures for holidays because they induce substantial carbon footprints (16% of total carbon footprint). This inclusive approach follows Druckman and Jackson, 2010, who estimate 10% of the carbon footprint in UK 2004 for holidays. Holidaymaking has no clear equivalent in time-use surveys, which cover mostly normal days. Furthermore, these activities are not restricted to formal holidays, but can occur on weekends, during short workweeks, or on special occasions even on a workday. Therefore, we allocated these footprints to free time activities outside of the home. It is important to show these expenses, as they will be spent probably on one or the other leisure activity if income is available and have therefore been pointed out as a potential source of rebound effects (Ottelin et al., 2014; Underwood and Fremstad, 2018). The ongoing discussion on higher footprints stemming from activities during holiday making (see Barr et al., 2010) cannot be analysed with the time-use approach as long as we lack enough data on holidaymaking.

### 5.2 Functional time-use analysis of demand-side potentials for decarbonisation

Analysing the functional time-use categories helps to perceive and analyse the different aims of re/production and offer different options for change towards low-carbon everyday life. For this purpose, we include all activities and all household expenditure in order to be able to see the total average carbon footprint per activity as well as per day. This offers a baseline for developing scenarios for changes in time-use patterns. Changing free time activities from Hobbies to Socializing for example would make a difference of 6% of daily carbon footprints, if the money saved is not spent otherwise. This example shows that the potential of reducing carbon footprints is closely linked to the question of time rebound (Hertwich 2005,Font Vivanco et al., 2016,) and still needs to be integrated in the analysis of low-carbon lifestyles (Schanes et al., 2016). Further research in time-use preferences could better inform calculations on plausible changes of carbon footprints of everyday life. In the following, we discuss some potentially interesting avenues for further research and time policies alike:

• **Person system**: personal time for sleep, care and eating is necessary for the personal reproduction. Having enough time for it is crucial for the personal well-being. This category includes necessary activities with quite high carbon footprints. Options for decarbonisation could consist in using less direct energy and long-lasting goods, as well as spending more time with personal activities and have to be analysed.
• **Household system**: caring time for others and all other activities, which are necessary for the reproduction of the household, can be reduced in terms of the amount of hours through adequate communal services (e.g. childcare facilities, meals in schools, day care for old persons), thus creating time for personal activities and lessening time squeeze. Carbon emissions could be reduced through the sharing of space, infrastructure and services in bigger households, new forms of shared living or sharing services. As the access to services can lead to increased use in turn (Frenken and Schor, 2017) further research on time use preferences is needed.
• **Economic system**: reducing working hours could alleviate time squeezes and enable spending more time with high quality of life and low-carbon activities in all other categories, for example with the family or in community activities or using less carbon intensive transport. Clearly, such reductions shouldn't lead to decreasing incomes below adequate levels (for further discussion, see: Wiedenhofer et al., 2018; Antal, 2018). Overall, such a measure could be advantageous for gender equality as well as for climate change mitigation.
• **Community system**: leisure activities mostly have a low carbon intensity, while few activities involve high costs and highest carbon emissions. Here we see the importance of the economic situation and prevailing unequal distribution and practices. Wealthy Austrians spent a large amount of money and thereby carbon emissions on holidays, catering and accommodation services. Whereas, leisure activities at home and at a short distance have the lowest carbon footprint. In this category, gender differences also become most pronounced. Communal, shared and attractive infrastructure for sports, entertainment, culture and participative activities could help to develop practices for low-carbon leisure activities irrespective of income. The lowest carbon footprint per hour comes from contributing to community and volunteering activities.
• The highest carbon footprint by far results from **mobility**. Mobility is not a sphere of re/production, but has to be assigned to the respective activities, which require or encompass a certain form of mobility. The footprint of mobility can be changed via 1) the need for mobility and 2) the length of distances to be covered and 3) the possibilities for modal split.

### 5.3 Gendered division of labour

In our analysis, we found significant differences in time use between an average woman and an average man. The traditional gendered division of labour leads generally to more time and money being available for men to spend on leisure activities and to a time squeeze for women when engaged in employment and caring, leading to higher resource use, if financially feasible (Eurostat, 2004; Ferguson, 2013; Ghassemi and Kronsteiner-Mann, 2009; McGinnity and Russell, 2007). Our results show a traditional gendered division of labour. The findings are similar to the results found by Druckman for UK 2005. In both countries emissions are higher for contracted activities for average women, mainly due to the higher amount of hours they spend with household and caring work and for free time activities for average men, mainly because they spend more time with free time activities. As caring and household activities take up more time for women than for men, the carbon footprint of these activities is allocated to them as well. Yet the benefits of these activities are for the household and not for the women themselves. Putting the load of household and caring activities with its carbon footprint on women is one of the methodological questions Druckman raises (Druckman et al., 2012).

These results constitute an important contribution to discussions about reducing and/or redistributing working hours equally among all adults.

Work-time reductions are discussed as an important measure for sustainable development. Buhl and Acosta have put forward three possible dividends of work-time reduction: 1) working less and having less income leads to lower consumption and therefore lower carbon footprints; 2) time can be spent with activities more favourable to life satisfaction; 3) sharing working hours among more people and spending more time with community activities leads to more equality and safer and inclusive cities (Buhl and Acosta, 2016). Additionally, a fourth dividend would be a higher degree of gender equality (Hartard et al., 2006).

These effects can be mobilized best if minimum wages secure an adequate level of income, workplaces are available (preferable within short commuting distance) and education/training as well as caring services are provided (Hayden, 1999; Knight et al., 2013; Nässen and Larsson, 2010; Pullinger, 2014; Schor, 2005; Shao and Rodríguez-Labajos, 2016). How the extra time resulting from work time reduction is spent is crucial for analysing these effects. We need further research on time use preferences among different demographic groups and the time rebound effects resulting from these preferences.

### 5.4 Desiderata for future research

The systemic approach including all time-use activities and carbon footprints of all carbon emissions of households provides a method, which can serve as a start for further analysis of rebound effects and options for low-carbon pathways. However, a number of constraints need to be addressed in further research. Ultimately, future research should aim to understand what can be learned for the necessary decarbonisation of everyday life, when going beyond consumer scapegoatism (Akenji, 2014), by taking time-use, societal norms about work and gender roles as well as infrastructural constraints and opportunities of households explicitly into account (Wiedenhofer et al., 2018).

Firstly, there is no objective way to decide which time-use activities meet basic human needs and which are optional, luxury or easier to change. For further analysis, time-use preferences, consumption patterns and their contributions to quality of life are required and differences among demographic and social groups have to be explicitly investigated, taking into account the politics of gentrification and low-carbon urbanization (Rice et al., 2019).

Secondly, to be able to differentiate between the impacts of time constraints and economic constraints, a clear direct link in time use and expenditure data is required. Currently, in Austria, both data sources are independently collected and no direct individual-level link can be made. Time-use data need to contain income information. Improved linkages between different surveys would therefore be required.

Thirdly, regular time-use surveys are necessary to enable longitudinal studies. Currently, the next wave of time-use surveys has been postponed due to political reasons and the socio-politically charged nature of insights into gender-differences.

Finally, distances to be covered, urban form, and the availability of infrastructure and services probably make an important difference in terms of time-use and consumption. Data including location of households and infrastructure could help to shed light on these interrelations.

## 6 Conclusions: time-use perspectives in sustainability research

Time-use surveys are increasingly used to monitor societal change and environmental implications along important issues such as urbanization, time squeeze, accelerated work-consumption cycles, gendered division of labour, and inequality across time and space (Aall et al., 2011; Brencic and Young, 2009; De Lauretis et al., 2017; Heinonen et al., 2013a; Jalas, 2005a, 2002; Jalas and Juntunen, 2015; Schipper et al., 1989; Yu et al., 2019).

With this work, we want to push the time-footprint frontier into more robust, reproducible and differentiated estimates. For this purpose, we have developed a systemic approach building on four conceptually grounded functional time-use categories and covering the direct and indirect emissions of household consumption. Clearly, this can only be a start and points to questions that should be investigated further (Wiedenhofer et al., 2018): How do time-use patterns, socio-economic conditions and consumption footprints interact and dynamically change over time? How do they vary between different household sizes and rural and urban areas? Are there other influencing factors? What are the major options and limits for change?

The functional time-use perspective applied herein provides the grounds to assess constraints and potentials of activity-based demand-side solutions to climate change mitigation. Time use required for the household and family as well as the economic system will strongly shape all other activities. Personal time cannot be compressed endlessly without serious health risks. These time-use constraints have to guide the concepts of scenarios for low-carbon everyday life. Developing these scenarios is an important next step to enable further analysis to discuss environmental implications of time policies. Conceptualizing everyday life in such a way has been conceptualized around practice theories and socio-technical provisioning systems (Ropke, 2015). Practices shape the way we are used to act in our daily routine and they are closely connected to communal infrastructure and provisioning systems.

The carbon intensity for singular activities calculated in this paper provide indications where the focus of demand-side climate change mitigation policies could be effective and highlights potential co-benefits in terms of personal well-being. The volume of carbon emissions results from the number of hours spent with a certain activity and the required consumption on goods and services for this activity. If only a short time is spent with high-carbon activities and long periods are spent with low-carbon activities, the total emissions can be changed to a significant extent. For example, multi-modal mobility strategies could focus on this. A short time spent in a car, with other people as passengers, followed by a longer trip using public means of transport and some walking or cycling to reach the destination, could save emissions and money and lower health risks (Haas, 2018). The large footprints due to cooking could be used for a shared meal with local products among more persons, who then spend longer leisure time together having fun. Everyone driving to an expensive restaurant and spending less time there, while inducing substantial indirect emissions along supply chains, would cause much higher emissions. Jalas describes the possibility of “making time” with activities, which take a long time but little material and energy, illustrating it with the “art of loving wooden boats” (Jalas, 2005b). Long-lasting goods lower the carbon emissions of daily use. Interestingly, watching TV as a habitual way of spending free time does not produce high emissions as long as we do not buy new devices frequently – however well-being effects should be critically considered. The same holds for music and books, where sharing has a long tradition through libraries. A library in short distance, the knowledge of how many interesting books can be found there, feasible opening hours, moderate prices and a routine to know about borrowing books add up to a reading practice with a much lower carbon footprint than, for example buying books online. However, subsequent shifts in potentially available incomes need to be taken into account, in order not to ignore the emissions rebound due to increased consumption of other goods and services. Clearly, these interesting avenues are worthy of further research.

A perceived time squeeze can also affect carbon footprints, for instance, through higher consumption of fast food or utilizing services perceived to be faster (e.g. dry cleaning, express deliveries). A high demand on ‘fast’ mobility also stems from pressures due to working hours, household/family related activities such as care obligations, or transporting children, older people or pets. Having more leisure time can lead to less time squeeze and lower emissions, by opening up the possibilities to accommodate low carbon activities. The amount of personal and free time is an indicator for time sovereignty. Spending it with high or low-carbon activities is strongly linked to available income and also shaped by societal norms on paid work, arrangements around care work in the family and the “attractiveness” of certain practices, for example long-distance tourism versus local/regional recreation. This points to the role of recreational infrastructure and public space within walking distance or reachable by carbon-free mobility, potentially changing practices of spending free time and all other daily activities.

However, this discussion points towards the most important desiderata for further research: For seriously assessing these options we need further research analysing differences in income, household size, urban form and residential location. Regular time-use surveys including these data and data on time-use preferences are urgently needed in order to be able to analyse effects of social change and policies over time.

Overall, we would argue that a change of perspectives away from “consume less and differently” towards “spend time with pleasant low carbon activities” could be used for a range of positive strategies towards demand-side measures for decarbonisation. Time policies (Reisch, 2015) have been positioned as a trans-sectional framework to find solutions for social and economic problems. Linking time use to carbon footprints yields new perspectives on the dilemma between human well-being, production and consumption and the environment (Creutzig et al., 2018; Lamb and Steinberger, 2017; Wiedenhofer et al., 2018). We would argue that a functional time-use perspective on consumption and footprints could contribute conceptually to questions of inclusive and multi-focal development goals (SDGs). Our approach shows how to widen the focus from consumption and money towards time and activity. This offers a tool to find ways of reducing carbon footprints of everyday activities, which can enhance quality of life and alleviate climate change at the same time.

## Declaration of Competing Interest

None.

## Appendix A Supplementary data

Supplementary tables Image 1
