# Supplementary material for: Time Matters: The Carbon Footprint of Everyday Activities in Austria
Source: Ecol Econ. 2019 Oct;164:106357. doi: 10.1016/j.ecolecon.2019.106357 (PMC6686204; doi:10.1016/j.ecolecon.2019.106357)

Supplementary Information 1: Time-Consumption Matrix: a 83 x 53 concordance matrix between Time-Use Activities and COICOP categories

Supplementary Information 2: CO2e intensity in kg/h per activity


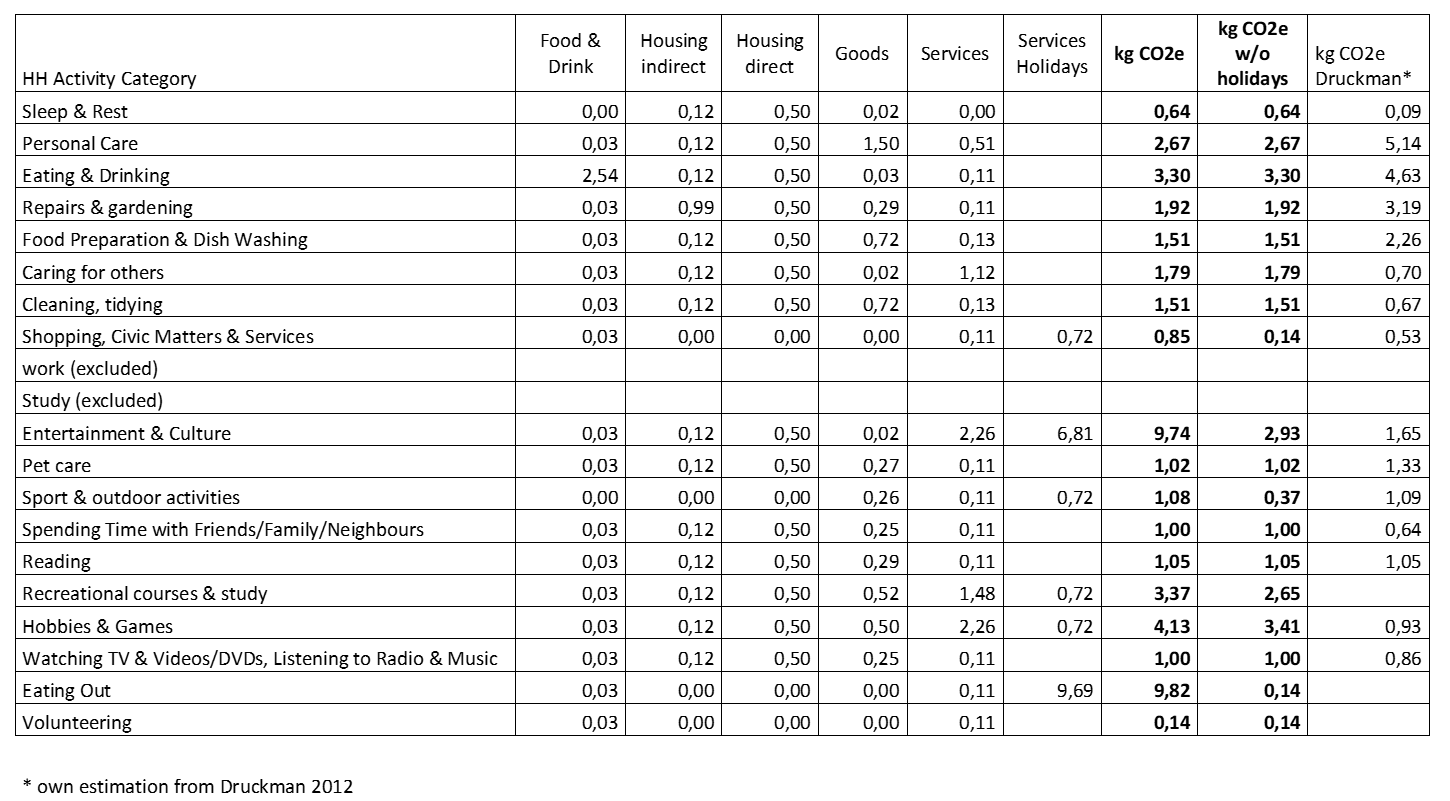

Supplement: Supplementary file 1 — Supplementary tables [file mmc1.docx]
